# GrainPest-SSL: A Lightweight Semi-Supervised Detector for Stored-Grain Pest Monitoring in Smart Granaries

**DOI:** 10.3390/s26144447

**Published:** 2026-07-13

**Authors:** Yanbo Chen, Xusheng Wei, Huanran Wei, Yuyao Jiang, Bo Mao

**Affiliations:** School of Computer Science and Artificial Intelligence, Nanjing University of Finance and Economics, Nanjing 210023, China; 2120231962@stu.nufe.edu.cn (Y.C.); 2120231765@stu.nufe.edu.cn (X.W.); 2120231424@stu.nufe.edu.cn (H.W.); 2120231368@stu.nufe.edu.cn (Y.J.)

**Keywords:** stored-grain pest detection, smart granary, semi-supervised object detection, pseudo-label purification, YOLOv8n-CAEMA, small-object detection, edge deployment

## Abstract

Reliable stored-grain pest monitoring is essential for smart granaries, yet probe-based field images pose three coupled bottlenecks: tiny and densely distributed pests in complex backgrounds, costly bounding-box annotation, and limited edge-side computing resources. To address these bottlenecks in a targeted manner, this study proposes GrainPest-SSL, an integrated framework comprising a field dataset, a lightweight detector, and a pseudo-label purification-based semi-supervised pipeline. First, to overcome the lack of realistic training data, a GrainPest dataset with 1000 field images and 21,676 annotated pest instances is constructed using multiple self-developed monitoring probes deployed in a large wheat flat granary, capturing systematic pest-monitoring images from different in-bin locations rather than a single fixed imaging point. Second, to improve small-target detection under resource constraints, a YOLOv8n-CAEMA detector is designed with a P2 detection head and tail-inserted Coordinate Attention (CA) and Efficient Multi-scale Attention (EMA), achieving 0.840 mAP@0.5 under full supervision with only 2.932 M parameters. Third, to reduce annotation dependence without adding inference-stage complexity, an offline Teacher–Student strategy with Pseudo-Label Purification Filtering (PPLF) refines pseudo-labels using confidence, size, and aspect-ratio priors; under the 30% labeled setting, GrainPest-SSL improves mAP@0.5 from 0.738 to 0.799 and mAP@0.5:0.95 from 0.322 to 0.369 on average over three random seeds. Comparisons with representative agricultural pest detectors and semi-supervised object detection (SSOD) methods further confirm the balanced accuracy–efficiency performance of GrainPest-SSL under label-limited conditions. The deployed Student detector further achieves 13.6 FPS in FP16 mode on a Jetson Orin Nano Dev Kit under the 10 W power mode, supporting scheduled pest inspection, early infestation screening, and intelligent warning in smart granary monitoring systems.

## 1. Introduction

Stored grain is an essential component of food security and agricultural supply-chain stability. During storage, insect pests can consume grain, contaminate products with excreta and fragments, reduce seed viability, and promote secondary microbial deterioration, thereby causing both quantitative and qualitative losses [1,2]. Wheat is an important stored grain commodity, and large flat granaries require continuous pest monitoring to support timely infestation-control decisions. In practical grain-condition monitoring systems, multiple sensing probes can be installed at different in-bin locations to capture pest activity and environmental status. Therefore, probe-based pest monitoring in large wheat flat granaries represents a realistic and operationally meaningful application scenario. Early and reliable pest monitoring is therefore critical for modern grain storage management. Conventional methods, such as manual visual inspection, probe sampling, sieving, and trap-based counting, are still widely used in practice [3,4]. However, these methods are usually labor-intensive, time-consuming, and dependent on expert experience, making it difficult to provide continuous and objective monitoring information in large-scale grain storage environments.

To improve the automation of stored-grain pest monitoring, various sensing techniques have been investigated, including acoustic detection, electronic-nose sensing, trap-based monitoring, and image-based recognition [5,6,7]. Among them, computer vision has attracted increasing attention because it can provide direct spatial information about pest appearance, number, and distribution. Early vision-based methods mainly relied on handcrafted features or traditional machine learning, which were sensitive to illumination, background impurities, insect pose, and image quality. With the rapid development of deep learning, convolutional neural networks and object detection models have been increasingly applied to stored-product insect detection and identification [8,9,10,11,12,13,14,15,16,17]. Recent YOLO-based studies, including several applications reported in agricultural sensing and smart monitoring scenarios, have shown promising performance in stored-grain pest detection, counting, and automated inspection [18,19,20,21]. Lightweight agricultural pest detectors such as Yolo-Pest and YOLO-LCE have also been proposed to improve detection efficiency in field and crop-monitoring scenarios [22,23].

Despite this progress, practical stored-grain pest detection still faces three coupled bottlenecks in probe-based smart granary monitoring. **Bottleneck 1: challenging field imagery.** Stored-grain pests usually appear as tiny and densely distributed targets in images captured by fixed monitoring probes, while grain residues, dust, uneven illumination, and complex backgrounds increase missed detections and false positives [18,19,20,24]. **Bottleneck 2: limited annotation resources.** Dense bounding-box annotation of small pest instances is laborious and requires domain expertise, making fully supervised learning difficult to scale in real storage management [8,9,10]. **Bottleneck 3: edge-side deployment constraints.** Monitoring terminals must balance detection accuracy with compact model size, stable training behavior, and predictable inference cost for periodic image-based inspection rather than continuous video analytics [25,26,27,28,29].

Existing studies partially address these issues but leave important gaps. Recent YOLO-based pest detectors mainly focus on architectural enhancement under fully supervised settings [18,19,20,21,22,23], while field datasets collected from operational grain storage probes remain limited. However, most existing pest detection methods still rely on fully supervised training, while few studies systematically evaluate semi-supervised detection under label-limited stored-grain monitoring conditions [30,31,32]. Advanced semi-supervised object detection methods such as Unbiased Teacher, Soft Teacher, and Dense Teacher improve label efficiency in general domains [33,34,35,36,37], but their online training pipelines are relatively complex and pseudo-label noise is still severe in dense small-object scenes [38,39,40]. Moreover, few studies jointly validate detection accuracy, label efficiency, and edge-side deployability within the same probe-based monitoring workflow.

To close these gaps in a problem–solution–evidence-aligned manner, this study proposes GrainPest-SSL, a lightweight offline semi-supervised detection framework for stored-grain pest monitoring. YOLOv8n is selected as the base detector because its anchor-free decoupled head, task-aligned label assignment, and mature open-source implementation provide stable training on small-scale field datasets and a practical accuracy–efficiency baseline for edge-side deployment [18,19,20,41,42]. On this basis, GrainPest-SSL integrates three components that directly correspond to the three bottlenecks above: (i) a field-oriented GrainPest dataset for realistic model development and evaluation; (ii) a YOLOv8n-CAEMA detector with a P2 detection head and tail-inserted CA/EMA modules for small-target representation under compact computational cost; and (iii) an offline Teacher–Student pipeline with Pseudo-Label Purification Filtering (PPLF) to exploit unlabeled monitoring images while suppressing background-induced pseudo-label noise through confidence-, size-, and aspect-ratio-guided filtering. The main contributions of this study are summarized as follows:

- A field-oriented GrainPest dataset is constructed using multiple self-developed grain pest monitoring probes deployed in a large wheat flat granary, containing 1000 images and 21,676 pest instances with dense small targets, grain residues, impurities, illumination variations, and local environmental differences across probe locations.
- A lightweight YOLOv8n-CAEMA detector is designed by integrating a P2 detection head and applying CA and EMA only at the tail of the C2f module. Under full supervision, it achieves 0.840 mAP@0.5 with 2.932 M parameters and 12.5 GFLOPs, selected as a balanced trade-off among Precision, mAP@0.5, compactness, and semi-supervised compatibility rather than as a uniformly best variant on every metric.
- A semi-supervised training strategy with PPLF is developed to reduce reliance on manual annotations without increasing inference-stage complexity. Under the 30% labeled setting, GrainPest-SSL improves mAP@0.5 from 0.738 to 0.799 and mAP@0.5:0.95 from 0.322 to 0.369 on average over three random seeds, achieving competitive performance against representative SSOD baselines under the same protocol, and the deployed Student detector achieves 13.6 FPS in FP16 mode on a Jetson Orin Nano Dev Kit under the 10 W power mode.

## 2. Materials and Methods

### 2.1. Dataset

#### 2.1.1. Monitoring Probe and Field Data Acquisition

To construct a dataset that reflects practical pest monitoring conditions in smart granaries, raw image data were collected using self-developed grain pest monitoring probes deployed in a large operational wheat flat granary. Unlike laboratory image acquisition, the collected images were obtained from an operational storage environment with practical disturbances such as grain residues, dust, uneven illumination, and complex backgrounds. Multiple probes were installed at different in-bin locations rather than at a single fixed imaging point, thereby covering local differences in grain-surface state, pest density, illumination, dust accumulation, and background interference. Although the dataset is collected from one granary and one grain type, the multi-probe deployment provides systematic field diversity beyond single-point laboratory acquisition. The monitoring probe was designed as an integrated sensing terminal for long-term field operation, integrating pest attraction, visual sensing, environmental monitoring, and wireless data transmission.

**Figure 1:**
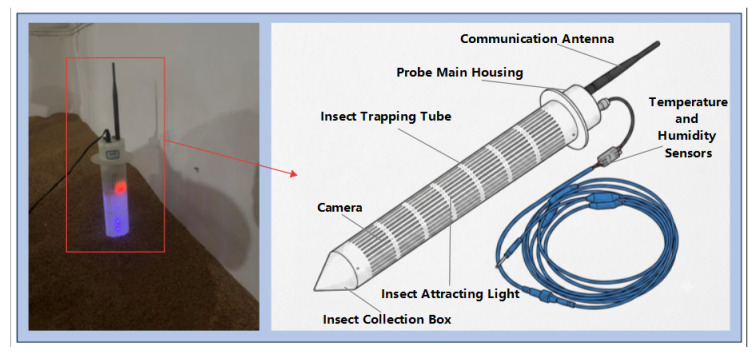
Field deployment example and structural composition of the self-developed grain pest monitoring probe. The left panel shows one probe deployed in a large wheat flat granary as a representative example, and the right panel illustrates its main components, including the communication antenna, probe main housing, insect trapping tube, camera, insect-attracting light, insect collection box, and temperature and humidity sensors. During data collection, multiple probes were installed at different in-bin locations for systematic pest monitoring; the GrainPest dataset was constructed from images captured by this multi-probe deployment scenario.

As shown in Figure 1, the left panel presents the field deployment of the probe inside the grain storage facility, while the right panel illustrates its main structural components. The probe mainly consists of a communication antenna, probe main housing, insect trapping tube, camera, insect-attracting light, insect collection box, temperature and humidity sensors, and a wireless communication module. The insect-attracting light guides stored-grain pests into the trapping and imaging area, where the imaging module periodically captures pest images according to a predefined acquisition interval. Meanwhile, the temperature and humidity sensors record environmental conditions inside the grain storage facility, providing auxiliary information for pest risk assessment.

The imaging unit of the probe was equipped with a MIS5001 5-MP camera module. According to the manufacturer’s specification, this module uses an MIS5001 image sensor with 5 million effective pixels, a 1/2.7-inch CMOS sensor size, and a maximum resolution of 2592 × 1944 pixels. Considering data transmission efficiency, storage cost, and the computational requirements of subsequent detection experiments, the captured images were stored at a resolution of 1280 × 720 pixels.

During field deployment, the probe was installed in an operational grain storage environment rather than a controlled laboratory environment. Therefore, the acquired images contain practical disturbances commonly observed in grain storage scenarios, such as grain residues, dust, impurities, insect fragments, uneven illumination, partial occlusion, and complex backgrounds. Long-term deployment may also introduce gradual image quality degradation caused by dust accumulation on the imaging window. In addition, stored-grain pests usually appear as small targets with similar appearances and diverse poses, making automatic detection more challenging. These sensor-captured images were used as the raw data source for constructing the GrainPest dataset.

#### 2.1.2. Data Annotation

A total of 1892 raw stored-grain pest images were initially collected using the monitoring probe. After quality control to remove blurred, incomplete, poorly illuminated, or indistinguishable samples, 1000 clean images were retained to construct the GrainPest dataset.

**Figure 2:**
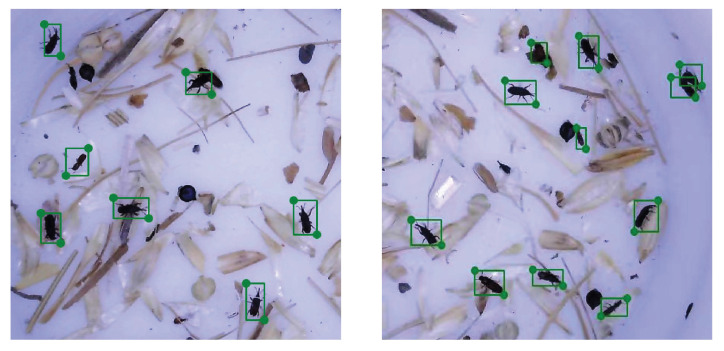
Manual annotation of stored-grain pest instances using LabelImg.

All visible pest instances were manually annotated using the open-source image annotation tool LabelImg, as shown in Figure 2. Since this study focuses on pest detection rather than species classification, all targets were assigned to a single category, namely, *pest*. This single-class setting is aligned with the primary operational goal of probe-based smart granary monitoring, namely, timely detection of pest presence, pest quantity, and activity level for early warning and fumigation decision support, rather than species-level diagnosis. Fine-grained pest-species recognition remains an important but separate future extension. In total, 21,676 pest instances were annotated in the 1000 images. The annotation results were saved in YOLO format, where each label file contains the class index and the normalized center coordinates, width, and height of each bounding box. After manual annotation, all labels were checked to reduce missing labels, inaccurate bounding boxes, and inconsistent annotations.

### 2.2. Overall Architecture of GrainPest-SSL

**Figure 3:**
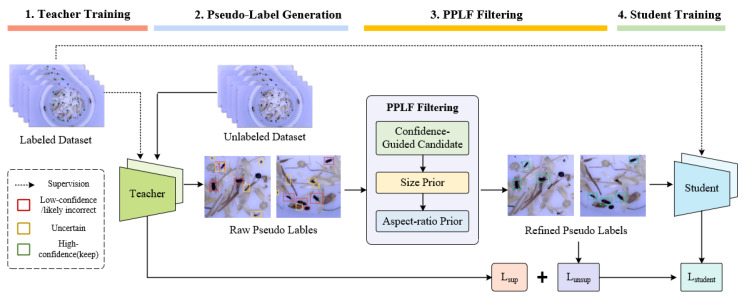
Overall architecture of the proposed GrainPest-SSL framework.

The proposed GrainPest-SSL framework is designed as a lightweight semi-supervised detection framework for stored-grain pest monitoring in smart granaries. It aims to reduce the dependence on large-scale expert annotations by leveraging both limited manually labeled images and abundant unlabeled monitoring images. As shown in Figure 3, the overall framework consists of four sequential stages: Teacher training, pseudo-label generation, Pseudo-Label Purification Filtering (PPLF), and Student training.

The workflow of GrainPest-SSL is described as follows:

- **Teacher training:** The Teacher model is first trained using the labeled subset of the training pool. The labeled images provide supervised learning signals, enabling the Teacher model to acquire initial stored-grain pest detection capability.
- **Pseudo-label generation:** The trained Teacher model is then used to perform inference on the unlabeled dataset. For each unlabeled image, the Teacher model generates raw pseudo-labels, which may contain high-confidence predictions, uncertain predictions, and low-confidence or incorrect predictions.
- **PPLF filtering:** To improve the reliability of pseudo-labels, the raw pseudo-labels are refined by the PPLF module. This module consists of confidence-guided candidate selection, size prior filtering, and aspect-ratio prior filtering. Low-quality predictions that are inconsistent with the visual characteristics of stored-grain pests are removed, while more reliable pseudo-labels are retained for Student training.
- **Student training:** The lightweight Student detector is trained using both labeled and unlabeled data. The labeled data provide the supervised loss, while the unlabeled data with PPLF-refined pseudo-labels provide the unsupervised pseudo-label loss. The final Student loss is obtained by combining these two learning signals.

During training, the Student detector is optimized by jointly using the supervised loss from labeled images and the unsupervised pseudo-label loss from unlabeled images. This design enables the Student model to learn from reliable pseudo-labels while reducing its dependence on large-scale manual annotations. During inference, only the lightweight Student detector is retained, whereas the Teacher model and PPLF module are discarded. Therefore, the final deployment stage requires only the compact Student detector with approximately 2.9 M parameters, which reduces computational overhead and facilitates efficient stored-grain pest monitoring on agricultural sensing probes or associated edge devices.

### 2.3. Lightweight Base Detector: YOLOv8n-CAEMA

**Figure 4:**
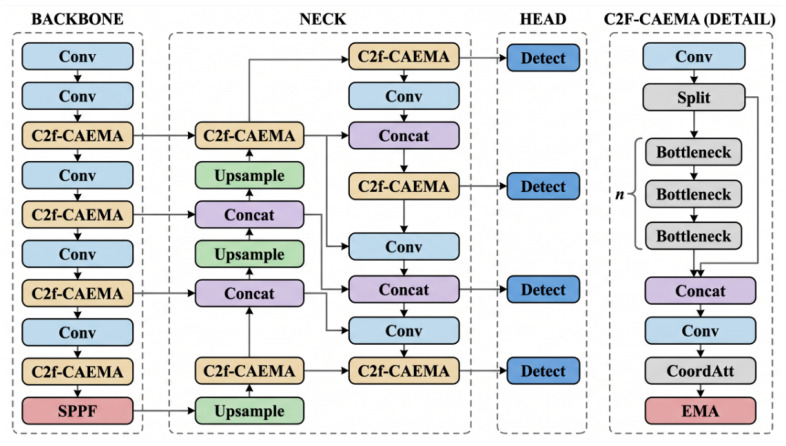
The overall architecture of the proposed YOLOv8n-CAEMA detector with the C2f-CAEMA module.

YOLOv8n-CAEMA is adopted as the lightweight base detector in GrainPest-SSL to capture tiny and densely distributed stored-grain pests. YOLOv8n is selected as the backbone because its anchor-free decoupled head and task-aligned label assignment provide stable optimization behavior on the present single-class and label-limited setting, while its open-source implementation facilitates fair comparison with recent YOLO-based pest detection studies [18,19,20,41,42]. As shown in Figure 4, YOLOv8n-CAEMA builds upon the YOLOv8n baseline by extending the FPN-PAN neck and introducing an additional high-resolution P2 detection head. Compared with the original three-scale detection design, the P2 branch preserves finer spatial details that are crucial for small pest localization [24,43,44,45,46]. The extended neck performs top-down feature fusion from P5 to P4, P3, and P2, followed by bottom-up feature aggregation from P2 to P3, P4, and P5. Finally, detection is performed on four scales, namely, Detect(P2), Detect(P3), Detect(P4), and Detect(P5).

The core component of this architecture is the C2f-CAEMA module. Based on the original C2f structure, the main topology of convolution, split, Bottleneck aggregation, concatenation, and output convolution is retained. To enhance feature representation with limited additional computational cost, Coordinate Attention (CA) [47] and Efficient Multi-scale Attention (EMA) [48] are sequentially inserted at the tail of the C2f module. Specifically, CA is first applied after the output convolution to encode directional spatial information and strengthen positional awareness, and EMA is then used to enhance multi-scale feature interactions. This tail-inserted CAEMA design improves the discriminative representation of small pest regions while avoiding repeated attention insertion inside each Bottleneck block.

Although the P2 detection head and the CAEMA attention path are introduced, YOLOv8n-CAEMA still maintains a compact model size of approximately 2.93 M parameters. This is mainly because the detector is designed for single-class pest detection, which substantially reduces the parameters of the classification branch compared with multi-class detection settings. Meanwhile, the additional P2 branch is implemented with lightweight channel configurations in the neck and detection head, so it mainly increases high-resolution feature computation rather than causing a proportional increase in model parameters. In addition, CA and EMA are inserted only at the tail of the C2f-CAEMA module and use lightweight compression and grouped feature interaction operations, instead of being repeatedly embedded inside each Bottleneck block. Therefore, the parameter reduction brought by the single-class detection head offsets the limited additional parameters introduced by the P2 branch and attention modules.

Thus, YOLOv8n-CAEMA improves small-target feature extraction through a lightweight P2-assisted multi-scale detection design and tail-inserted attention enhancement. It serves as a suitable base detector for GrainPest-SSL, enabling the Student model to balance detection accuracy and deployment efficiency in smart granary pest monitoring scenarios.

### 2.4. Offline Teacher-Student Self-Training Strategy

The proposed GrainPest-SSL framework adopts an offline Teacher–Student self-training strategy. As described in Section 2.2, the Teacher model is first trained using the labeled subset and then kept fixed to generate pseudo-labels for unlabeled images, avoiding the exponential moving average updates used in online Mean Teacher methods. This offline pseudo-labeling strategy provides a stable and reproducible pseudo-label generation process, while the PPLF module further refines the generated pseudo-labels before Student training.

Let $D_{L}={\left\{\left(x_{i}^{l},y_{i}^{l}\right)\right\}}_{i=1}^{N_{L}}$ and $D_{U}={\left\{x_{j}^{u}\right\}}_{j=1}^{N_{U}}$ denote the labeled and unlabeled datasets, respectively, where $x_{i}^{l}$ is a labeled image, $y_{i}^{l}$ is its ground-truth annotation, and $x_{j}^{u}$ is an unlabeled image. The fixed pretrained Teacher detector $f_{T}\left(\cdot \right)$ generates raw pseudo-labels ${\tilde{y}}_{j}^{u}=f_{T}\left(x_{j}^{u}\right)$ for unlabeled images. These raw predictions are refined by the PPLF module, ${\hat{y}}_{j}^{u}=G\left({\tilde{y}}_{j}^{u}\right)$, where G(·) denotes the pseudo-label purification operation. The refined pseudo-labels ${\hat{y}}_{j}^{u}$ are then used as pseudo-supervisory signals for Student training.

The Student detector $f_{S}\left(\cdot \right)$ is trained using both labeled images and unlabeled images with refined pseudo-labels. Since the proposed framework does not modify the original YOLOv8 loss formulation, the overall Student training objective can be expressed as

(1) $$L_{student}=L_{sup}+\lambda L_{unsup},$$

where $L_{sup}$ denotes the supervised loss computed on labeled data, $L_{unsup}$ denotes the unsupervised loss computed on unlabeled data with refined pseudo-labels, and λ is the weighting coefficient for balancing the two loss terms. In this study, λ was empirically set to 1.0 in all experiments.

For labeled images, the supervised loss is defined as

(2) $$L_{sup}=\frac{1}{N_{L}}\sum_{i=1}^{N_{L}}\left[L_{box}\left(f_{S}\left(x_{i}^{l}\right),y_{i}^{l}\right)+L_{cls}\left(f_{S}\left(x_{i}^{l}\right),y_{i}^{l}\right)+L_{dfl}\left(f_{S}\left(x_{i}^{l}\right),y_{i}^{l}\right)\right].$$

Similarly, the unsupervised loss for unlabeled images is defined as

(3) $$L_{unsup}=\frac{1}{N_{U}}\sum_{j=1}^{N_{U}}\left[L_{box}\left(f_{S}\left(x_{j}^{u}\right),{\hat{y}}_{j}^{u}\right)+L_{cls}\left(f_{S}\left(x_{j}^{u}\right),{\hat{y}}_{j}^{u}\right)+L_{dfl}\left(f_{S}\left(x_{j}^{u}\right),{\hat{y}}_{j}^{u}\right)\right].$$

Here, $L_{box}$, $L_{cls}$, and $L_{dfl}$ represent the bounding-box regression loss, classification loss, and distribution focal loss, respectively. The term $f_{S}\left(\cdot \right)$ represents the network predictions, including classification scores, bounding-box coordinates, and distributional regression outputs, and the above loss terms correspond to the standard YOLOv8 loss implementation. After training, only the Student detector is retained for inference, while the Teacher model and PPLF module are used only during offline training.

### 2.5. Pseudo-Label Purification Filtering

To reduce the negative impact of noisy pseudo-labels generated by the offline Teacher model, a Pseudo-Label Purification Filtering (PPLF) module is introduced. The PPLF operator G(·) refines the raw pseudo-labels ${\tilde{y}}_{j}^{u}$ through a task-oriented filtering pipeline, aiming to retain reliable pseudo-labels for Student training. Specifically, PPLF consists of three sequential steps: confidence-guided candidate selection, size prior filtering, and aspect-ratio prior filtering.

#### 2.5.1. Confidence-Guided Candidate Selection

The raw pseudo-labels generated by the Teacher model may contain low-confidence predictions and redundant overlapping boxes caused by grain residues, impurities, shadows, or complex background interference. Therefore, a confidence-guided candidate selection process, incorporating confidence thresholding and non-maximum suppression (NMS), is first applied.

For each raw pseudo box $b\in {\tilde{y}}_{j}^{u}$ with confidence score $c_{b}$, low-confidence predictions are first filtered out to form an intermediate candidate set:

(4) $$B_{conf}=\left\{b|c_{b}\geq \tau_{c},\;b\in {\tilde{y}}_{j}^{u}\right\},$$

where $\tau_{c}$ denotes the confidence threshold. In this study, $\tau_{c}$ was set to 0.50 to maintain relatively high pseudo-label precision. The default PPLF thresholds were derived from the statistical distribution of ground-truth bounding boxes in the labeled training subset rather than from test-set tuning; a dedicated sensitivity analysis is reported in Section 3.4.3.

Subsequently, standard NMS is applied to $B_{conf}$ to eliminate redundant detections around the same pest target. Boxes having an Intersection over Union (IoU) greater than the predefined threshold $\tau_{iou}$ with a higher-scoring box are suppressed. The resulting candidate set is defined as

(5) $$B_{c}=NMS\left(B_{conf},\tau_{iou}\right),$$

where $\tau_{iou}$ was set to 0.45 in this study. The candidate set $B_{c}$ is then used as the input for subsequent task-specific prior filtering.

#### 2.5.2. Size Prior Filtering

Since the images in the GrainPest dataset were captured by a fixed monitoring probe under relatively stable imaging conditions, stored-grain pest targets usually fall within a reasonable bounding-box size range after resizing to the unified network input scale. In contrast, extremely small or abnormally large boxes are more likely to correspond to background fragments, grain impurities, incomplete targets, or false detections. Therefore, size prior filtering is introduced to remove pseudo boxes with abnormal areas.

For each candidate box $b\in B_{c}$, its normalized area is calculated as

(6) $$A\left(b\right)=w_{b}\times h_{b},$$

where $w_{b}$ and $h_{b}$ denote the normalized width and height of the bounding box, respectively. The size-filtered candidate set is defined as

(7) $$B_{s}=\left\{b|S_{min}\leq A\left(b\right)\leq S_{max},\;b\in B_{c}\right\},$$

where $S_{min}$ and $S_{max}$ denote the lower and upper bounds of the valid pest bounding-box area, respectively. In this study, $S_{min}$ and $S_{max}$ were set to 0.0003565 and 0.0037227, respectively, corresponding to approximately 146 and 1525 pixels under the 640 × 640 input scale. The specific threshold values $\left[S_{min},S_{max}\right]$ were empirically determined based on the statistical distribution of the ground-truth bounding boxes in the labeled dataset $D_{L}$.

#### 2.5.3. Aspect-Ratio Prior Filtering

In addition to bounding-box size, the aspect ratio of pest targets provides useful task-specific prior information. In probe-captured stored-grain pest images, valid pest boxes usually follow a relatively stable shape distribution, whereas boxes with abnormal aspect ratios are more likely to be caused by background edges, insect fragments, overlapping impurities, or inaccurate predictions. Therefore, aspect-ratio prior filtering is further applied after size prior filtering.

For each candidate box $b\in B_{s}$, to avoid direction bias and treat horizontally and vertically oriented pest targets consistently, the aspect ratio is calculated as the ratio of the longer side to the shorter side:

(8) $$R\left(b\right)=\max\left(\frac{w_{b}}{h_{b}},\frac{h_{b}}{w_{b}}\right).$$

The final refined pseudo-label set is obtained as

(9) $$B_{final}=\left\{b|1.0\leq R\left(b\right)\leq R_{max},\;b\in B_{s}\right\},$$

where $R_{max}$ denotes the upper bound of the valid aspect-ratio range. In this study, $R_{max}$ was set to 3.89. The specific threshold value $R_{max}$ was empirically determined based on the statistical shape distribution of the ground-truth bounding boxes in the labeled dataset $D_{L}$.

Finally, the refined pseudo-labels are expressed as

(10) $${\hat{y}}_{j}^{u}=G\left({\tilde{y}}_{j}^{u}\right)=B_{final}.$$

Compared with confidence-guided candidate selection alone, the proposed PPLF module further incorporates task-specific size and aspect-ratio priors from the stored-grain pest monitoring scenario. This design removes pseudo boxes that pass confidence thresholding and NMS but are inconsistent with the expected spatial scale or shape characteristics of pest targets, thereby providing more reliable pseudo-supervisory signals for Student training.

## 3. Results

### 3.1. Experimental Setup

The experimental platform consisted of an Intel Core i9-14900KF 24-core processor, 64 GB DDR5 4800 MHz memory, and an NVIDIA GeForce RTX 5070 Ti GPU with 16 GB video memory. The software environment included Windows OS, Python 3.10, and PyTorch 2.12.0 with CUDA 13.0. All experiments were conducted using an Ultralytics-based YOLO implementation.

Before being fed into the network, all images were resized to a unified input size of 640 × 640 during training and inference. During training, the maximum number of epochs was set to 300, with an early-stopping patience of 50 epochs. The Adam optimizer was adopted with an initial learning rate of 0.001. The batch size was set to 4, and the number of workers was set to 0. Additionally, cache loading was disabled and automatic mixed precision (AMP) training was enabled to streamline the training process and ensure reproducible optimization. No offline augmented images were generated; the standard online augmentation strategy of the YOLO training pipeline was retained during training.

#### Dataset Partition and Semi-Supervised Protocol

To evaluate the proposed GrainPest-SSL framework under label-limited conditions, the 1000 clean images in the GrainPest dataset were divided into training, validation, and test sets at a ratio of 7:2:1. Specifically, 700 images were used as the training pool, 200 images were used for validation, and 100 images were used for testing. The validation and test sets were kept fixed throughout all experiments to ensure a fair and consistent evaluation.

To simulate the label-scarcity problem caused by the limited availability of domain experts in real smart granary monitoring scenarios, different label proportions were adopted within the training pool. Specifically, 10%, 20%, 30%, and 50% of the 700 training images were randomly selected as labeled data for initial Teacher model training, while the remaining images were treated as unlabeled data for pseudo-label-based Student model training. Although the unlabeled images had corresponding manual annotations in the complete dataset, these annotations were not used during semi-supervised training.

To reduce sampling bias, each label-proportion setting was repeated using three random seeds, namely, 2026, 2027, and 2028, and the average performance was reported. The detailed distribution of labeled and unlabeled images under each setting is summarized in Table 1.

### 3.2. Evaluation Metrics

Precision, Recall, mAP@0.5, and mAP@0.5:0.95 were used as the main accuracy metrics for the single-class GrainPest dataset. In the following tables, Precision and Recall are abbreviated as P and R, respectively. Precision measures the proportion of correctly detected pest targets among all predictions, while Recall measures the proportion of correctly detected pest targets among all ground-truth targets:

(11) $$\mathrm{Precision}=\frac{\mathrm{TP}}{\mathrm{TP}+\mathrm{FP}}$$

(12) $$\mathrm{Recall}=\frac{\mathrm{TP}}{\mathrm{TP}+\mathrm{FN}}$$

where TP, FP, and FN denote true positives, false positives, and false negatives, respectively.

mAP@0.5 denotes the average precision at an IoU threshold of 0.50:

(13) $$\mathrm{mAP}@0.5=AP_{0.50}$$

mAP@0.5:0.95 is a stricter metric that averages AP values over IoU thresholds from 0.50 to 0.95 with a step size of 0.05:

(14) $$\mathrm{mAP}@0.5:0.95=\frac{1}{10}\sum_{t=0.50}^{0.95}AP_{t}$$

where $AP_{t}$ denotes the average precision at the IoU threshold *t*.

Model compactness and deployment efficiency were evaluated using the number of parameters, GFLOPs, inference latency, and frames per second (FPS). FPS was calculated as

(15) $$\mathrm{FPS}=\frac{1000}{\mathrm{Latency}}$$

where Latency denotes the end-to-end inference time per image in milliseconds.

### 3.3. Overall Performance Under Different Label Proportions

**Figure 5:**
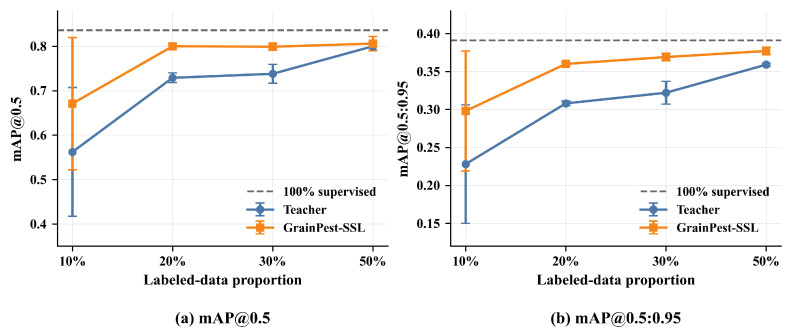
Performance comparison under different labeled-data proportions. (**a**) mAP@0.5 comparison. (**b**) mAP@0.5:0.95 comparison. Error bars indicate standard deviations over three random seeds, and the dashed horizontal line denotes the 100% fully supervised YOLOv8n-CAEMA upper bound.

To evaluate the effectiveness of GrainPest-SSL under label-limited conditions, experiments were conducted under 10%, 20%, 30%, and 50% labeled-data proportions. For each proportion, three random seeds (2026, 2027, and 2028) were used, and the results are reported as mean ± standard deviation. The Teacher model trained only with labeled data was used as the baseline. GrainPest-SSL exploits unlabeled images by generating pseudo-labels offline using a fixed Teacher detector and refining them with PPLF before Student training. A 100% fully supervised YOLOv8n-CAEMA model was included as the upper-bound reference. The results are summarized in Table 2 and Figure 5.

GrainPest-SSL improved mAP@0.5 across all labeled-data proportions. Under the 10% labeled setting, mAP@0.5 increased from 0.562 for the Teacher baseline to 0.671 for GrainPest-SSL. Under the 20%, 30%, and 50% settings, GrainPest-SSL achieved mAP@0.5 values of 0.800, 0.799, and 0.806, respectively. As shown in Figure 5a, the 50% labeled result approached the 100% fully supervised upper bound of 0.836, indicating that the proposed framework can recover strong detection performance with substantially fewer manual annotations.

The improvements were also consistent under the stricter mAP@0.5:0.95 metric. GrainPest-SSL increased mAP@0.5:0.95 from 0.228 to 0.298 under the 10% labeled setting, corresponding to a relative improvement of 30.7%. Under the 20%, 30%, and 50% settings, the relative improvements were 16.9%, 14.6%, and 5.0%, respectively. As shown in Figure 5b, the mAP@0.5:0.95 results indicate that PPLF-refined pseudo-labels also contribute to improved detection performance under stricter IoU thresholds.

Precision and Recall further support the effectiveness of pseudo-label purification. Under the 10%, 20%, and 30% settings, GrainPest-SSL improved Precision from 0.552 to 0.666, from 0.675 to 0.772, and from 0.691 to 0.744, respectively, suggesting that PPLF helps reduce false positives from noisy pseudo-labels. Recall also improved under all labeled-data proportions. Although Precision slightly decreased under the 50% setting, Recall, mAP@0.5, and mAP@0.5:0.95 still improved, indicating a better overall detection trade-off.

The relatively large error bars under the 10% setting indicate that the model is more sensitive to random sampling when very few labeled images are available. With only 70 labeled training images, different random seeds can produce labeled subsets with substantially different pest density, background complexity, and Teacher initialization quality. Because the offline Teacher is fixed before pseudo-label generation, this initial Teacher-quality fluctuation is directly propagated to the Student model, making the 10% setting highly volatile. This behavior highlights a practical trade-off between annotation cost and model stability: although semi-supervised learning still improves the average performance at 10% labeling, deployment-oriented smart granary systems should preferably maintain at least 20–30% labeled data for more stable training. In contrast, the 50% setting leaves less room for semi-supervised improvement because the Teacher baseline already benefits from a larger labeled subset. The 30% labeled setting therefore provides a practical middle ground: it still reflects an annotation-limited scenario while offering more stable evaluation than the 10% setting. For this reason, the 30% labeled setting was used for the subsequent ablation, comparison, and sensitivity analyses.

### 3.4. Ablation Study

To further evaluate the effectiveness of the key components in GrainPest-SSL, ablation experiments were conducted from two aspects: the detector architecture and the pseudo-label purification strategy. First, the detector ablation was performed to justify the selection of YOLOv8n-CAEMA as the base detector. Then, the PPLF ablation was conducted to analyze the contribution of pseudo-label purification to Student training.

#### 3.4.1. Ablation Study on the YOLOv8n-CAEMA Detector

Before analyzing the pseudo-label purification strategy, an ablation study was conducted on the detector architecture to justify the selection of YOLOv8n-CAEMA as the base detector of GrainPest-SSL. This experiment was performed under the fully supervised setting with seed 2026, so that the effect of detector design could be evaluated independently from the semi-supervised training process. Considering that stored-grain pests are usually small and densely distributed, the effects of the P2 detection head, Coordinate Attention (CA), and Efficient Multi-scale Attention (EMA) were evaluated under the same training and testing protocol. The results are shown in Table 3.

As shown in Table 3, the P2 detection head improves Recall from 0.800 to 0.832, mAP@0.5 from 0.820 to 0.839, and mAP@0.5:0.95 from 0.378 to 0.387. This indicates that the high-resolution P2 branch is beneficial for detecting small pest targets by preserving finer spatial details. Based on the P2-enhanced detector, CA further improves mAP@0.5:0.95 to 0.394, suggesting better localization under stricter IoU thresholds, while EMA increases Precision to 0.777, indicating improved discriminative feature representation.

The complete YOLOv8n-CAEMA detector achieves the highest Precision of 0.787 and the highest mAP@0.5 of 0.840 among the evaluated variants, while maintaining a compact model size of 2.932 M parameters and a moderate computational cost of 12.5 GFLOPs. By contrast, YOLOv8n+P2+CA achieves the highest mAP@0.5:0.95 of 0.394, which is 0.003 higher than that of YOLOv8n-CAEMA. Therefore, YOLOv8n-CAEMA was selected not as a uniformly best model across all metrics, but as the variant offering the most suitable overall trade-off among Precision, mAP@0.5, compactness, and compatibility with the subsequent semi-supervised framework.

**Figure 6:**
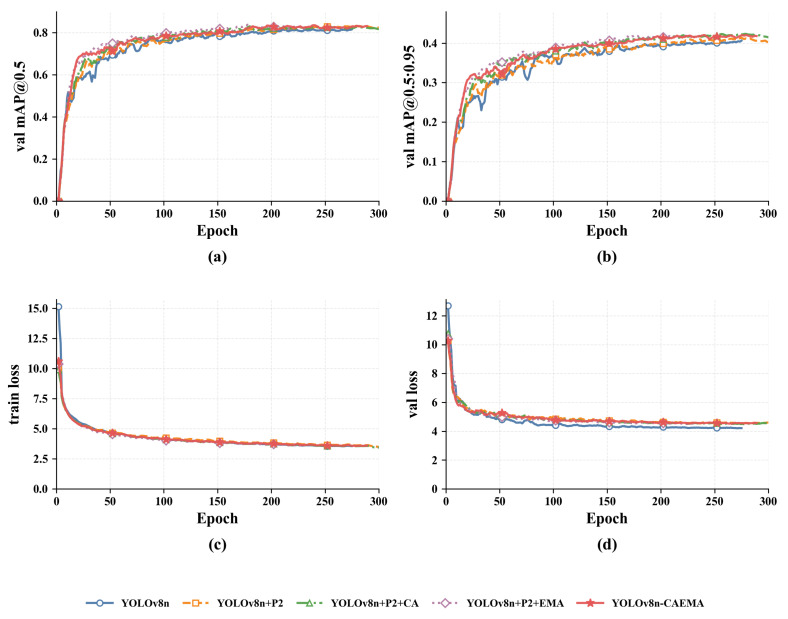
Training dynamics of different YOLOv8n-CAEMA detector variants. Distinct line styles and markers are used for black-and-white readability. (**a**) Validation mAP@0.5. (**b**) Validation mAP@0.5:0.95. (**c**) Training loss. (**d**) Validation loss.

Figure 6 shows the training dynamics of different detector variants, including validation mAP curves and loss curves. The P2-enhanced and attention-enhanced models generally converge to higher validation mAP than the YOLOv8n baseline, especially under the stricter mAP@0.5:0.95 metric. Meanwhile, the train-loss and validation-loss curves decrease smoothly and gradually stabilize, indicating that the added P2 head and attention modules do not introduce obvious training instability. These curves are used to illustrate convergence behavior and optimization stability, while the final quantitative comparison is based on the test-set results in Table 3.

With the detector architecture fixed, the following subsection further analyzes the contribution of PPLF to pseudo-label purification.

#### 3.4.2. Ablation Study on the PPLF Module

With YOLOv8n-CAEMA fixed as the base detector, an ablation study was conducted to evaluate the contribution of the proposed Pseudo-Label Purification Filtering (PPLF) module. The experiment was performed under the 30% labeled setting with seed 2026. Here, confidence-guided candidate selection refers to confidence thresholding followed by non-maximum suppression (NMS). The results are shown in Table 4.

As shown in Table 4, Teacher-only serves as the supervised baseline because it does not use unlabeled images, achieving 0.726 mAP@0.5 and 0.305 mAP@0.5:0.95. Directly using raw Teacher pseudo-labels does not reliably improve Student performance. Although Raw Self-Training obtains a high PL-R of 0.9554, its PL-P is only 0.1037, indicating severe pseudo-label noise with many false positives. Consequently, its mAP@0.5 drops to 0.701, showing that noisy pseudo-labels can degrade Student learning.

Confidence-guided candidate selection is the key filtering step. It increases PL-P from 0.1037 to 0.9365 and improves mAP@0.5:0.95 from 0.314 to 0.358, confirming that removing low-confidence and redundant boxes is essential for reliable pseudo-supervision. The geometric priors further refine the selected pseudo-labels. Compared with confidence-guided selection alone, adding the size prior improves mAP@0.5:0.95 to 0.366. To isolate the effect of shape constraints, a confidence-guided selection + aspect-ratio prior variant was evaluated, achieving 0.362 mAP@0.5:0.95. Compared with the size-prior variant, this result indicates that scale and shape priors provide complementary geometric constraints for filtering background clutter.

The full PPLF module achieves the best overall detection performance, with the highest mAP@0.5 of 0.794 and mAP@0.5:0.95 of 0.369. These results also indicate that pseudo-label Precision and Recall are not linearly translated into final detection accuracy. For example, Raw Self-Training has very high PL-R but low PL-P, which introduces many false positives and degrades Student performance, whereas overly strict filtering may improve pseudo-label reliability but reduce supervisory coverage. Full PPLF provides a more effective balance between pseudo-label reliability and coverage, even though it does not obtain the highest PL-P or PL-R individually. Compared with Teacher-only, Full PPLF improves mAP@0.5:0.95 from 0.305 to 0.369, corresponding to a relative gain of 21.0%. These results demonstrate that PPLF can convert noisy Teacher predictions into more useful pseudo-supervisory signals for Student training under label-limited conditions.

#### 3.4.3. Sensitivity Analysis of PPLF Thresholds

To evaluate whether PPLF depends on narrowly tuned manual thresholds, sensitivity experiments were conducted under the 30% labeled setting with seed 2026. First, with the default size and aspect-ratio priors fixed, only the confidence threshold $\tau_{c}$ was varied. As shown in Table 5, increasing $\tau_{c}$ from 0.30 to 0.70 raises pseudo-label Precision (PL-P) from 0.7745 to 0.9795 but reduces pseudo-label Recall (PL-R) from 0.6180 to 0.0977. Although lower thresholds such as 0.30 and 0.40 can occasionally yield slightly higher test mAP on this split, their PL-P values remain relatively low and may increase the risk of noisy pseudo-label accumulation. The default threshold $\tau_{c}=0.50$ therefore provides a conservative trade-off rather than single-metric optimality.

Second, with $\tau_{c}=0.50$ and NMS IoU = 0.45 fixed, loose, default, and strict settings were evaluated for the size prior and aspect-ratio prior independently. As shown in Table 6, PL-P remains stable within 0.9367–0.9401 across all geometric settings, indicating that pseudo-label purification does not rely on an extremely narrow threshold point. Strict settings reduce PL-R and final detection performance, whereas the default percentile-based thresholds derived from labeled-box statistics provide a more stable and interpretable choice.

### 3.5. Comparison with YOLO Detectors and Edge Deployment

#### 3.5.1. Comparison with Representative YOLO Detectors

**Figure 7:**
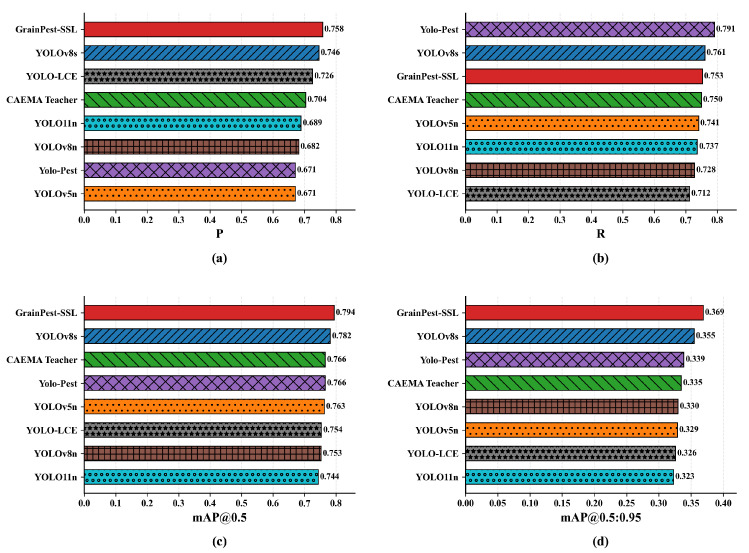
Performance visualization of GrainPest-SSL and representative YOLO-based detectors under the 30% labeled setting with seed 2026. (**a**) Precision. (**b**) Recall. (**c**) mAP@0.5. (**d**) mAP@0.5:0.95. GrainPest-SSL achieves the highest mAP@0.5 and mAP@0.5:0.95 while retaining the same inference-stage detector scale as the YOLOv8n-CAEMA Teacher, indicating that the observed accuracy gain mainly comes from the proposed semi-supervised learning strategy rather than a larger deployment model.

After the detector ablation in Table 3 established YOLOv8n-CAEMA as the compact base detector for GrainPest-SSL, a further comparison was conducted to evaluate whether the complete semi-supervised framework provides a better accuracy–complexity trade-off than representative YOLO-based detectors and recent agricultural pest detectors. GrainPest-SSL was compared with YOLOv5n, YOLOv8n, YOLO11n, YOLOv8s, Yolo-Pest [22], YOLO-LCE [23], and the supervised YOLOv8n-CAEMA Teacher. Yolo-Pest and YOLO-LCE were reproduced according to their published architectural descriptions and retrained under the same GrainPest protocol. All experiments were conducted under the 30% labeled setting with seed 2026; multi-seed averages are reported separately in Table 2. The quantitative results and their visual comparison are shown in Table 7 and Figure 7, respectively.

As shown in Table 7 and Figure 7, GrainPest-SSL achieves the best mAP@0.5 and mAP@0.5:0.95 among the compared detectors under the 30% labeled setting. Yolo-Pest obtains the highest Recall of 0.791 but the lowest Precision of 0.671 among the compared methods, while requiring 7.876 M parameters and 20.5 GFLOPs. YOLO-LCE is the most compact baseline with 1.651 M parameters and 5.5 GFLOPs, but its mAP@0.5 and mAP@0.5:0.95 remain lower than those of GrainPest-SSL, suggesting that excessive compression may weaken dense small-target representation in probe-captured pest images. Compared with the supervised YOLOv8n-CAEMA Teacher, GrainPest-SSL improves mAP@0.5 from 0.766 to 0.794 and mAP@0.5:0.95 from 0.335 to 0.369. Since the deployed Student detector has the same inference structure and parameter size as YOLOv8n-CAEMA, the improvement mainly comes from semi-supervised learning with purified pseudo-labels rather than additional inference-stage complexity. Compared with YOLOv8n and YOLO11n, GrainPest-SSL also achieves higher mAP@0.5 and mAP@0.5:0.95 under the same 30% labeled setting.

Compared with the larger YOLOv8s model, GrainPest-SSL achieves higher mAP@0.5 and mAP@0.5:0.95 while using only 2.932M parameters and 12.5 GFLOPs, which are much lower than the 11.134 M parameters and 29.0 GFLOPs of YOLOv8s. Although YOLO11n has the lowest computational cost of 6.7 GFLOPs, its mAP@0.5:0.95 is lower than those of YOLOv8n and GrainPest-SSL, suggesting that lower complexity alone does not guarantee better pest detection performance. The final Student detector maintains 192.3 FPS on the RTX 5070 Ti; this result is used as a same-platform inference reference, while practical edge deployment is evaluated on the Jetson Orin Nano Dev Kit in the following subsection.

#### 3.5.2. Comparison with Representative SSOD Methods

To ensure a fair and controlled comparison, representative SSOD strategies were adapted to the same YOLOv8n-CAEMA detector and evaluated under the same labeled/unlabeled data split and test set. This setup focuses the evaluation on the semi-supervised learning strategies themselves within the GrainPest scenario.

To further evaluate label efficiency, GrainPest-SSL was compared with representative semi-supervised object detection (SSOD) methods, including Soft Teacher, Unbiased Teacher, and Dense Teacher, under the same GrainPest data partition, 30% labeled setting, and seed 2026. All methods used the same training, validation, and test splits, and Teacher-only supervised training was included as the baseline. The results are summarized in Table 8.

Table 8 shows that all SSOD methods improve mAP@0.5 and mAP@0.5:0.95 over Teacher-only supervision, confirming that unlabeled monitoring images provide useful complementary supervision under label-limited conditions. GrainPest-SSL achieves the highest mAP@0.5 of 0.794 and the highest Recall of 0.753, indicating better pest-target completeness in dense probe-captured scenes. Unbiased Teacher obtains the highest mAP@0.5:0.95 of 0.371, which is 0.002 higher than that of GrainPest-SSL, suggesting a slight localization advantage under stricter IoU thresholds. Compared with Soft Teacher and Dense Teacher, GrainPest-SSL improves mAP@0.5:0.95 by 0.011 and 0.027, respectively. These results indicate that GrainPest-SSL offers a competitive and balanced SSOD performance while using a simpler offline Teacher–Student pipeline with task-specific pseudo-label purification rather than complex online Teacher updating.

#### 3.5.3. Edge Deployment Performance

To verify the practical edge-side applicability of GrainPest-SSL, the final Student detector was deployed on the Jetson Orin Nano Dev Kit under the 10 W power mode. Since the Teacher model and PPLF module are used only during offline training, only the Student detector was retained during deployment. The deployment results are shown in Table 9.

As shown in Table 9, the final Student detector achieves 12.4 FPS with 80.6 ms latency in FP32 mode and 13.6 FPS with 73.4 ms latency in FP16 mode on the Jetson Orin Nano Dev Kit. The corresponding energy efficiencies are 1.77 FPS/W and 2.03 FPS/W, respectively. These results show that the deployed Student detector maintains stable detection performance under different numerical precision settings. More importantly, stored-grain pest monitoring is usually performed through scheduled image capture and periodic inspection rather than continuous high-frame-rate video analysis. Therefore, the obtained Jetson performance is practically sufficient for edge-side pest screening, warning support, and routine storage-management inspection on resource-constrained monitoring devices. In the current smart granary workflow, only the Student detector is retained at the edge terminal, while the Teacher model and PPLF module remain offline training components and do not increase on-device computational burden. The detection module has been integrated into probe-based monitoring terminals for periodic pest-image analysis, pest-count estimation, activity-level screening, and fumigation-timing support together with temperature and humidity sensing.

### 3.6. Qualitative Detection Results

**Figure 8:**
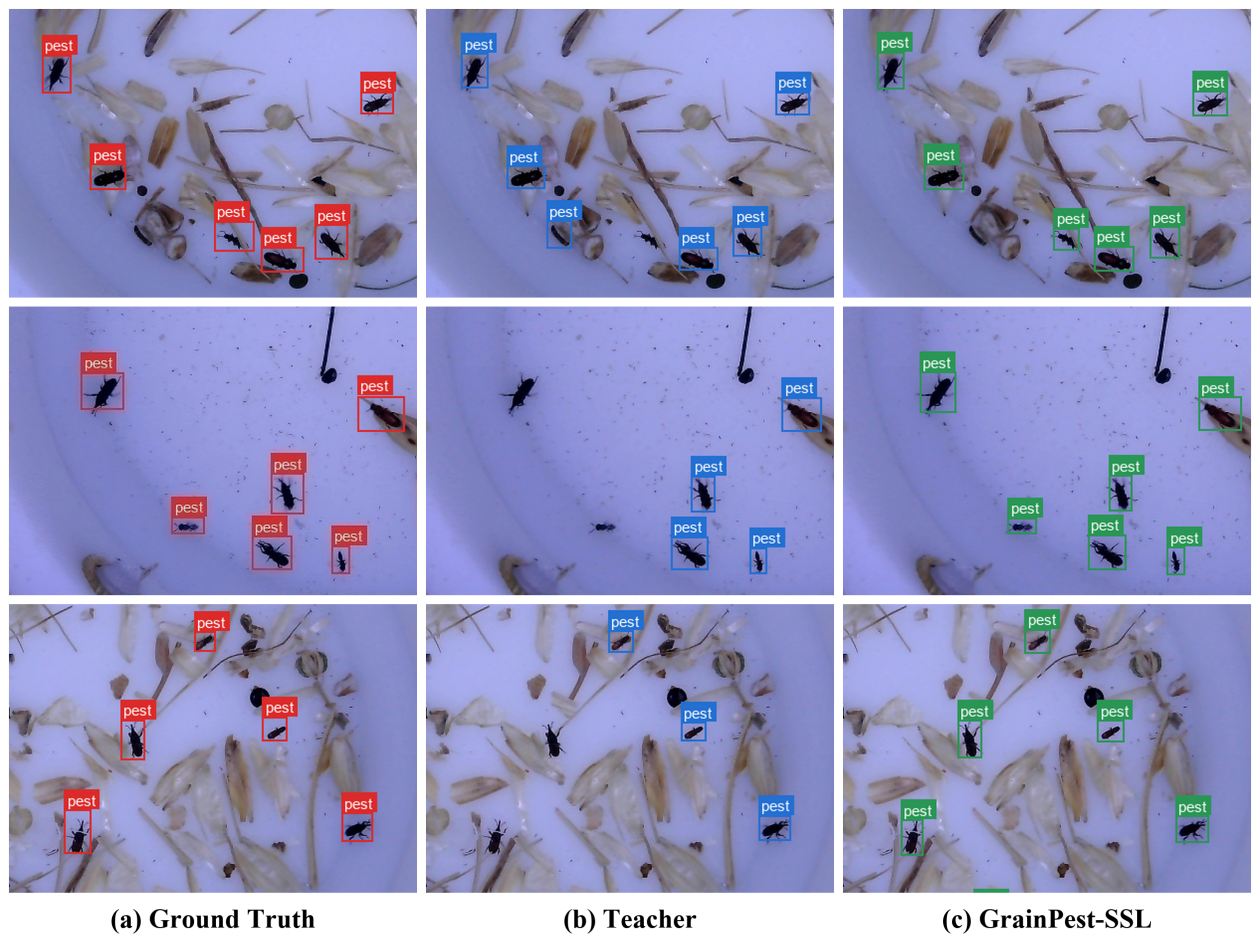
Enlarged qualitative detection results in low-density and high-density stored-grain pest monitoring scenes. Subplot titles inside the panels were removed and larger fonts were used for improved readability. (**a**) Ground-truth annotations. (**b**) Teacher predictions. (**c**) GrainPest-SSL Student predictions. Each panel is displayed at an enlarged scale to highlight the differences in missed detections and false positives between the Teacher baseline and the proposed Student detector.

Figure 8 presents enlarged qualitative detection results in low-density and high-density stored-grain pest monitoring scenes to facilitate visual comparison. The three columns show (a) ground-truth annotations, (b) Teacher predictions, and (c) GrainPest-SSL Student predictions, respectively. In low-density scenes, the GrainPest-SSL Student detector identifies most visible pest targets and provides more complete detection results than the Teacher baseline. Nevertheless, a few false detections still occur in challenging background regions, indicating that grain residues, impurities, and visually similar background objects may interfere with the detector.

In high-density scenes, pest instances are densely distributed and partially mixed with broken grains, chaff, and overlapping pest bodies. Under these conditions, the Teacher model tends to miss several small or crowded pest targets. In contrast, the GrainPest-SSL Student detector detects more pest instances and produces results closer to the ground-truth annotations. These visual comparisons demonstrate that the proposed semi-supervised framework improves detection completeness in both sparse and dense pest-monitoring scenarios. A detailed interpretation of these findings, together with literature comparisons, practical implications, and remaining limitations, is provided in Section 4.

## 4. Discussion

This section summarizes the main findings, compares GrainPest-SSL with existing methods, discusses its practical value in smart granary systems, and outlines the main limitations and future research directions.

### 4.1. Main Findings

The experimental results demonstrate that GrainPest-SSL effectively addresses label-limited stored-grain pest detection under probe-based monitoring constraints. First, the multi-probe GrainPest dataset provides realistic field imagery with dense small targets, background impurities, and local environmental differences across in-bin probe locations. Second, YOLOv8n-CAEMA improves small-target representation under compact complexity, although it is selected as a balanced detector rather than a uniformly best variant on every metric. Third, PPLF-guided offline semi-supervised training converts noisy Teacher predictions into more reliable pseudo-supervisory signals, enabling the Student detector to exploit unlabeled monitoring images without increasing inference-stage complexity. Under the 30% labeled setting, GrainPest-SSL improves average mAP@0.5 from 0.738 to 0.799 and mAP@0.5:0.95 from 0.322 to 0.369 over three random seeds.

### 4.2. Comparison with Existing Methods

Compared with representative YOLO detectors and agricultural pest detectors under the same 30% labeled protocol, GrainPest-SSL achieves the highest mAP@0.5 and mAP@0.5:0.95 while maintaining a compact 2.932 M-parameter Student model. Yolo-Pest yields higher Recall but lower Precision and larger computational cost, whereas YOLO-LCE is more compact but less effective for dense small pest targets in probe-captured images. Compared with stored-grain pest detection studies such as those based on FCA-YOLO and PDA-YOLO [19,20], the present work additionally evaluates label efficiency and edge-side deployment within the same probe-based workflow, although direct metric comparison across different datasets is not applicable.

Compared with representative SSOD methods, GrainPest-SSL achieves the highest mAP@0.5 and Recall, while Unbiased Teacher obtains a slightly higher mAP@0.5:0.95. This indicates that GrainPest-SSL is not uniformly superior on every SSOD metric, but it offers a strong overall balance between detection completeness, pseudo-label reliability, and deployment simplicity. The offline PPLF design is particularly suitable for resource-constrained monitoring terminals that require reproducible training and Student-only inference.

### 4.3. Practical Implications for Smart Granary Systems

From an application perspective, GrainPest-SSL is designed for periodic probe-image inspection rather than continuous video analytics. The final Student detector achieves 13.6 FPS in FP16 mode on a Jetson Orin Nano Dev Kit, which is sufficient for scheduled pest screening and early warning in smart granary monitoring workflows. Because only the Student detector is deployed at the edge, the semi-supervised training components do not increase on-device computational burden. In the current monitoring workflow, probe-based terminals perform periodic image acquisition, pest-count estimation, and activity-level screening, while temperature and humidity sensing provides auxiliary information for fumigation-timing support. The present single-class detection setting matches the operational need for early infestation warning based on pest presence and quantity, even though species-level recognition remains necessary for more targeted intervention strategies.

### 4.4. Limitations and Future Work

Several limitations should be acknowledged. First, although the GrainPest dataset is collected from a real large wheat flat granary using multiple in-bin probes, it is still limited to one granary type and one grain commodity; cross-granary, cross-grain-type, and cross-season validation have not yet been completed. Second, all insects are labeled as a single *pest* class, which limits species-specific management decisions. Third, PPLF uses statistically derived but fixed thresholds; although sensitivity analysis shows stable behavior on GrainPest, recalibration may be required when transferring to new camera setups or storage environments. Fourth, under the 10% labeled setting, performance remains sensitive to random seed selection because the offline Teacher quality fluctuates strongly when labeled data are critically scarce. Future work will extend the dataset to more granaries, grain types, and pest categories; investigate adaptive or learnable pseudo-label filtering; and explore online SSOD strategies and cross-site generalization.

## 5. Conclusions

This study targets three coupled bottlenecks in probe-based stored-grain pest monitoring—challenging field imagery, limited annotation resources, and edge-side deployment constraints—and addresses them through a problem–solution–evidence-aligned framework called GrainPest-SSL.

For **Bottleneck 1**, a field-oriented GrainPest dataset with 1000 images and 21,676 pest instances was constructed from a large wheat flat granary using multiple in-bin monitoring probes, providing realistic multi-location field data for algorithm development and validation. For **Bottleneck 2**, the detector-level contribution is YOLOv8n-CAEMA, which combines a P2 detection head with tail-inserted CA and EMA modules to improve small-target representation under compact complexity; under full supervision, it achieved 0.840 mAP@0.5 with 2.932 M parameters and 12.5 GFLOPs. For **Bottleneck 3**, the learning-strategy contribution is PPLF-guided offline semi-supervised training, which enables the Student detector to exploit unlabeled monitoring images through confidence-, size-, and aspect-ratio-guided pseudo-label refinement. During deployment, only the Student detector is retained, so the semi-supervised component improves training effectiveness without introducing additional inference-stage overhead.

The experimental evidence supports this alignment across label-scarce settings. GrainPest-SSL consistently improved detection performance under 10%, 20%, 30%, and 50% labeled-data proportions. Under the 30% labeled setting, it improved mAP@0.5 from 0.738 to 0.799 and mAP@0.5:0.95 from 0.322 to 0.369 on average over three random seeds, achieving competitive performance against representative agricultural pest detectors and SSOD baselines under the same protocol. Edge deployment on the Jetson Orin Nano Dev Kit further showed that the final Student detector achieves 13.6 FPS in FP16 mode under the 10 W power mode, which is suitable for scheduled pest-image inspection and early warning support rather than continuous video surveillance.

Future work will extend the current framework toward multi-granary and multi-grain-type validation, fine-grained pest-species recognition, adaptive pseudo-label filtering, and cross-site generalization.

## Figures and Tables

**Table 1:** Distribution of images in the GrainPest dataset under different label proportions.

Label Proportion	Labeled Images	Unlabeled Images	Validation Set	Test Set
10%	70	630	200	100
20%	140	560	200	100
30%	210	490	200	100
50%	350	350	200	100

**Table 2:** Overall performance comparison under different labeled-data proportions.

Label Ratio	Method	P	R	mAP@0.5	mAP@0.5:0.95
10%	Teacher	0.552 ± 0.139	0.632 ± 0.031	0.562 ± 0.145	0.228 ± 0.078
10%	GrainPest-SSL	0.666 ± 0.117	0.649 ± 0.047	0.671 ± 0.149	0.298 ± 0.079
20%	Teacher	0.675 ± 0.017	0.715 ± 0.038	0.729 ± 0.011	0.308 ± 0.003
20%	GrainPest-SSL	0.772 ± 0.007	0.740 ± 0.010	0.800 ± 0.006	0.360 ± 0.002
30%	Teacher	0.691 ± 0.027	0.748 ± 0.025	0.738 ± 0.021	0.322 ± 0.015
30%	GrainPest-SSL	0.744 ± 0.025	0.780 ± 0.029	0.799 ± 0.008	0.369 ± 0.005
50%	Teacher	0.761 ± 0.010	0.779 ± 0.020	0.800 ± 0.009	0.359 ± 0.002
50%	GrainPest-SSL	0.757 ± 0.023	0.790 ± 0.008	0.806 ± 0.016	0.377 ± 0.005
100%	Fully supervised	0.766 ± 0.022	0.811 ± 0.019	0.836 ± 0.011	0.391 ± 0.005

Note: P and R denote Precision and Recall, respectively.

**Table 3:** Ablation study of the YOLOv8n-CAEMA detector under the fully supervised setting with seed 2026.

Model	Params(M)	GFLOPs	P	R	mAP@0.5	mAP@0.5:0.95
YOLOv8n	3.006	8.1	0.760	0.800	0.820	0.378
YOLOv8n+P2	2.921	12.2	0.762	0.832	0.839	0.387
YOLOv8n+P2+CA	2.939	12.2	0.756	0.823	0.832	**0.394**
YOLOv8n+P2+EMA	2.923	12.4	0.777	0.793	0.832	0.389
YOLOv8n-CAEMA (Ours)	2.932	12.5	**0.787**	0.789	**0.840**	0.391

Note: P and R denote Precision and Recall, respectively. Bold values indicate the best result within each metric column. YOLOv8n-CAEMA denotes the final detector adopted in GrainPest-SSL, which integrates the P2 detection head, Coordinate Attention (CA), and Efficient Multi-scale Attention (EMA).

**Table 4:** Ablation study of the PPLF module under the 30% labeled setting with seed 2026.

Method	PL-P	PL-R	mAP@0.5	mAP@0.5:0.95
Teacher-only	–	–	0.726	0.305
Raw Self-Training	0.1037	0.9554	0.701	0.314
Confidence-guided selection	0.9365	0.3324	0.790	0.358
+ Size prior	0.9376	0.3296	0.790	0.366
+ Aspect-ratio prior	0.9367	0.3298	0.791	0.362
**Full PPLF (Ours)**	**0.9378**	**0.3321**	**0.794**	**0.369**

Note: PL-P and PL-R denote pseudo-label Precision and Recall, respectively. Full PPLF consists of confidence-guided candidate selection, size prior filtering, and aspect-ratio prior filtering, as defined in Section 2.5.

**Table 5:** Sensitivity analysis of the PPLF confidence threshold under the 30% labeled setting with seed 2026.

$\tau_{c}$	PL-P	PL-R	P	R	mAP@0.5	mAP@0.5:0.95
0.30	0.7745	0.6180	0.725	0.775	0.794	0.372
0.40	0.8772	0.4723	0.729	0.794	0.808	0.360
0.50	0.9378	0.3321	0.758	0.753	0.794	0.369
0.60	0.9653	0.2044	0.731	0.748	0.774	0.350
0.70	0.9795	0.0977	0.727	0.734	0.752	0.338

Note: P and R denote Precision and Recall, respectively. PL-P and PL-R denote pseudo-label Precision and Recall, respectively. Size and aspect-ratio priors were fixed at their default settings.

**Table 6:** Sensitivity analysis of PPLF geometric priors under the 30% labeled setting with seed 2026.

Prior	Setting	Range	PL-P	PL-R	P	R	mAP@0.5	mAP@0.5:0.95
Size	Loose	area [0.5, 99.5] aspect [1, 99]	0.9367	0.3297	0.763	0.769	0.812	0.374
Size	Default	area [1, 99] aspect [1, 99]	0.9378	0.3321	0.758	0.753	0.794	0.369
Size	Strict	area [2, 98] aspect [1, 99]	0.9401	0.3286	0.737	0.737	0.773	0.357
Aspect	Loose	area [1, 99] aspect [0.5, 99.5]	0.9376	0.3313	0.744	0.778	0.801	0.366
Aspect	Default	area [1, 99] aspect [1, 99]	0.9378	0.3321	0.758	0.753	0.794	0.369
Aspect	Strict	area [1, 99] aspect [2, 98]	0.9379	0.3254	0.773	0.754	0.794	0.360

Note: P and R denote Precision and Recall, respectively. PL-P and PL-R denote pseudo-label Precision and Recall, respectively. Range denotes the percentile bounds used for the size prior (area) and aspect-ratio prior (aspect). Confidence threshold and NMS IoU were fixed at 0.50 and 0.45, respectively.

**Table 7:** Comparison with representative YOLO detectors under the 30% labeled setting with seed 2026.

Method	Params(M)	GFLOPs	P	R	mAP@0.5	mAP@0.5:0.95	FPS
YOLOv5n	2.503	7.1	0.671	0.741	0.763	0.329	238.1
YOLOv8n	3.008	8.2	0.682	0.728	0.753	0.330	238.1
YOLO11n	2.592	6.7	0.689	0.737	0.744	0.323	227.3
YOLOv8s	11.134	29.0	0.746	0.761	0.782	0.355	217.4
Yolo-Pest	7.876	20.5	0.671	0.791	0.766	0.339	115.9
YOLO-LCE	1.651	5.5	0.726	0.712	0.754	0.326	82.9
YOLOv8n-CAEMA Teacher	2.932	12.5	0.704	0.750	0.766	0.335	185.2
GrainPest-SSL (Ours)	2.932	12.5	**0.758**	0.753	**0.794**	**0.369**	192.3

Note: P and R denote Precision and Recall, respectively. Bold values indicate the best result within each metric column. FPS was measured on an NVIDIA GeForce RTX 5070 Ti as a same-platform inference reference. During inference, only the Student detector is retained in GrainPest-SSL. Yolo-Pest and YOLO-LCE were architecture-reproduced and retrained under the unified GrainPest protocol.

**Table 8:** Comparison of representative SSOD strategies under the same YOLOv8n-CAEMA detector, 30% labeled setting, and seed 2026.

Method	P	R	mAP@0.5	mAP@0.5:0.95
Teacher-only	0.661	0.739	0.726	0.305
Soft Teacher	0.755	0.721	0.792	0.358
Unbiased Teacher	0.740	0.720	0.786	**0.371**
Dense Teacher	0.766	0.733	0.784	0.342
GrainPest-SSL (Ours)	0.758	**0.753**	**0.794**	0.369

Note: P and R denote Precision and Recall, respectively. Bold values indicate the best result within each metric column. All SSOD baselines were evaluated under the same labeled/unlabeled partition and fixed test set.

**Table 9:** Deployment performance of the final GrainPest-SSL Student detector on the Jetson Orin Nano Dev Kit.

Prec. Mode	P	R	mAP@0.5	mAP@0.5:0.95	Lat. (ms)	FPS	Power (W)	FPS/W
FP32	0.741	0.771	0.790	0.360	80.6	12.4	7.00	1.77
FP16	0.746	0.763	0.788	0.362	73.4	13.6	6.73	2.03

Note: P, R, Prec. Mode, Lat. and Power denote Precision, Recall, precision mode, latency, and average power, respectively. All tests were conducted on the Jetson Orin Nano Dev Kit under the 10 W power mode with an input size of 640 and batch size of 1. FPS/W denotes frames per second per watt.

## Data Availability

The data presented in this study are available from the corresponding author upon reasonable request.

## Abbreviations

The following abbreviations are used in this manuscript:

SSL	Semi-supervised learning
SSOD	Semi-supervised object detection
PPLF	Pseudo-label purification filtering
PL-P	Pseudo-label precision
PL-R	Pseudo-label recall
NMS	Non-maximum suppression
CA	Coordinate attention
EMA	Efficient multi-scale attention
IoU	Intersection over union
mAP	Mean average precision
FPS	Frames per second
GFLOPs	Giga floating-point operations
